# Three-Dimensional Geometric Deformation of the 12-Lead Electrocardiogram in Bundle Branch Block

**DOI:** 10.7759/cureus.117611

**Published:** 2026-10-07

**Authors:** Patrick Dumitrescu, Igor Antonio Narciso, Johnny Cobas Hernandez, Renato Gustavo Luque

**Affiliations:** 1 Internal Medicine Residency Program Lee Health, Florida State University College of Medicine, Cape Coral, USA

**Keywords:** 12-lead ecg, bundle branch block, cardiac conduction, electrocardiography, machine learning, ptb-xl, spatial geometry

## Abstract

Background: The 12-lead electrocardiogram (ECG) represents cardiac electrical activity as separate scalar leads, whereas ventricular activation is spatial and time-varying. We evaluated an interpretable experimental three-dimensional (3D) mathematical embedding of the 12-lead ECG, termed the ECG road, and quantified deformation of the ventricular depolarization complex (QRS) relative to a normal reference atlas.

Methods: PTB-XL (Physikalisch-Technische Bundesanstalt - Extra Large) version 1.0.3 (National Metrology Institute of Germany, Braunschweig, Germany) was partitioned using its official patient-safe folds (training folds 1-8, validation fold 9, and locked test fold 10). Strict normal ECGs were compared with clean complete left bundle branch block (CLBBB), complete right bundle branch block (CRBBB), and intraventricular conduction delay (IVCD). A QRS-focused normal road atlas was built exclusively from training normals. Four prespecified geometric features measured QRS trajectory scale and normalized Euclidean and Mahalanobis deformation. L2-regularized logistic models using demographics, geometry, or both were frozen before test evaluation. A QRS-duration-only analysis was retained as a secondary sensitivity benchmark. A post hoc morphology-aware conventional 12-lead comparator incorporating QRS duration and prespecified lead-level QRS morphology features was separately specified, trained on the training folds only, and frozen before test performance was evaluated. Confidence intervals (CIs) used 2,000 patient-level stratified bootstrap replicates.

Results: All 1,001 test ECGs were processed successfully for the primary geometry analysis. After deterministic selection of one ECG per patient, binary test cohorts contained 33 CLBBB cases among 867 patients, 21 CRBBB cases among 855 patients, and 26 IVCD cases among 860 patients; each comparison included 834 strict normals. The primary combined model achieved an area under the receiver operating characteristic curve (AUROC) of 0.9998 (95% CI 0.9992-1.0000) and average precision (AP) of 0.9951 (0.9833-1.0000) for CLBBB, and an AUROC of 0.9995 (0.9984-1.0000) and AP of 0.9858 (0.9546-1.0000) for CRBBB. IVCD discrimination was limited (AUROC 0.5857, 0.4725-0.6982; AP 0.0443, 0.0311-0.0852). In its own evaluable test cohort, the morphology-aware conventional comparator achieved AUROC/AP values of 1.0000/1.0000 for CLBBB, 0.9998/0.9860 for CRBBB, and 0.6614/0.0935 for IVCD.

Conclusions: The experimental 3D ECG representation preserved information sufficient to discriminate canonical CLBBB and CRBBB but not heterogeneous IVCD. Conventional lead-level morphology showed near-ceiling discrimination for canonical bundle branch block, and paired analyses did not demonstrate a clear performance advantage for either representation. These findings support the ECG road as an interpretable proof-of-concept mathematical representation of conduction-pattern information already present in the 12-lead ECG, rather than evidence of clinical superiority over conventional ECG criteria. External validation against independent clinical or imaging endpoints and established ECG-to-XYZ approaches is required before clinical application.

## Introduction

The surface 12-lead electrocardiogram (ECG) remains one of the most widely used cardiovascular diagnostic tests. Conventional interpretation separates the signal into lead-specific amplitudes, intervals, axes, and morphologic patterns. Ventricular depolarization is physiologically three-dimensional (3D) and time-varying, whereas the representation evaluated here is a mathematical transformation of the surface ECG rather than a direct reconstruction of the underlying electrical field. Consensus ECG criteria for bundle branch block (BBB) depend on the duration of the ventricular depolarization complex (QRS) together with characteristic lead morphologies that reflect delayed and redirected activation [1].

Vectorcardiography historically addressed this spatial problem by representing cardiac electrical forces as 3D loops [2]. Although direct Frank vectorcardiographic recording is uncommon in contemporary practice, validated transformations can synthesize orthogonal representations from the standard 12-lead ECG [3-5], and modern work has renewed interest in spatial QRS and QRS-T descriptors [6-8]. At the same time, deep learning has achieved strong ECG classification performance on large public datasets such as PTB-XL [9-11]; however, high predictive accuracy does not itself provide a compact geometric description of the abnormal activation pattern [12,13]. 

We therefore developed a complementary representation in which each lead amplitude is mapped at each time point to a predefined 3D lead direction, generating 12 spatial points and an instantaneous centroid trajectory, termed the ECG road. This research embedding is intentionally distinct from a validated Frank vectorcardiographic transformation: it is a mathematical coordinate system for studying trajectory deformation, not a claim of direct cardiac dipole reconstruction. The broader framework supports time-resolved road geometry, local principal-component structure, and 12-point constellation metrics; the present study focuses on a prespecified QRS road-deformation panel as the first locked proof-of-concept application. 

BBB was selected because canonical left and right BBB should provide an interpretable test of whether a normal-atlas deformation approach detects organized changes in ventricular activation. We hypothesized that complete left BBB (CLBBB) and complete right BBB (CRBBB) would show large QRS road deformation relative to strict normal ECGs, whereas the more heterogeneous intraventricular conduction delay (IVCD) category would be less separable. We further sought to determine whether geometric features contributed information beyond age and sex under a patient-exclusive, prospectively locked validation and test workflow. This prespecified objective evaluated incremental information relative to demographic variables and was not intended to establish superiority over established conventional ECG criteria.

## Materials and methods

Study design and data source

This was a retrospective computational study using PTB-XL (Physikalisch-Technische Bundesanstalt - Extra Large) version 1.0.3 (National Metrology Institute of Germany, Braunschweig, Germany), a publicly available collection of 21,799 10-second clinical 12-lead ECGs from 18,869 patients [9,10]. Only the 500-Hz recordings were used to maintain a uniform sampling rate and the temporal resolution used for QRS-energy anchoring and time-resolved geometric analysis. Because all 21,799 PTB-XL records had an available 500-Hz waveform representation, use of the 500-Hz signals did not exclude any ECG records from the source dataset. PTB-XL provides recommended stratification folds designed to support reproducible algorithm development and evaluation [9-11]. We used folds 1-8 for training, fold 9 for held-out validation, and fold 10 for locked test evaluation. Patient identifiers were explicitly checked across partitions, and no patient identifier was shared between training, validation, and test sets.

The primary cohort contained 9,904 ECGs from 9,216 patients. At the ECG level, training contained 7,908 records, validation 995, and test 1,001. The test set comprised 42 CLBBB, 22 CRBBB, 26 IVCD, and 911 strict-normal ECGs. The locked test gate confirmed 1,001 records from 914 unique patients before waveform processing.

This secondary analysis used publicly available, de-identified data; the original dataset publication describes the ethical governance of PTB-XL [9]. PTB-XL version 1.0.3 is available through PhysioNet [10]. Analysis was developed in Google Colab (Google LLC, Mountain View, California, United States) using Python 3.13.15 (Python Software Foundation, Wilmington, Delaware, United States) with NumPy 2.1.3, pandas 2.2.3, SciPy 1.16.3, Matplotlib 3.10.0, WFDB 4.3.1, scikit-learn 1.6.1, and joblib 1.5.3, with a fixed random seed of 42; frozen configuration, environment records, and artifact hashes were retained. 

Diagnostic cohort definitions

A strict normal was defined as a diagnostic superclass set containing only NORM. Standard communications protocol for computer-assisted electrocardiography (SCP) diagnostic statements were considered positive only when their score was greater than zero. Clean conduction cohorts were defined as CLBBB, CRBBB, or IVCD while excluding concomitant myocardial infarction, ST/T change, and hypertrophy superclasses. Other bundle-branch groups were excluded from each binary target-versus-normal model. These choices intentionally created a proof-of-concept phenotype set with reduced diagnostic overlap rather than an all-comers clinical differential-diagnosis cohort.

Three-dimensional lead-vector embedding

Each ECG lead was assigned a fixed 3D unit vector representing its research embedding direction. At each time point, the processed voltage from each lead was used as a scalar weight on that lead's embedding vector. In other words, the spatial position for each lead was obtained by multiplying the processed lead amplitude by that lead's fixed embedding vector. The 12 resulting spatial points formed a time-indexed lead constellation, and the mean position of this constellation defined the ECG road used for downstream geometric analysis.

Coordinates were defined as x toward the patient's left, y inferiorly, and z anteriorly. Limb-lead directions in the x-y plane were I at 0 degrees, II at +60 degrees, III at +120 degrees, aVR at -150 degrees, aVL at -30 degrees, and aVF at +90 degrees. The initial research embedding placed V1-V6 in the x-z plane at 140, 115, 90, 60, 30, and 0 degrees, respectively.

The precordial directions are an experimental research embedding and are not validated physical lead fields. Accordingly, orientation-sensitive raw roads were preserved; no per-record principal component analysis rotation or Kabsch alignment was used in the locked primary atlas analysis, and future sensitivity testing with established ECG-to-Frank or ECG-to-XYZ transformations is required [3-5].

Signal preprocessing and quality control

Raw waveforms were retained unchanged. Baseline estimation used sequential 200-ms and 600-ms median filters. The primary geometry signal was band-pass filtered from 0.5 to 40 Hz with zero-phase Butterworth filtering; a 0.5-100 Hz broadband representation was also retained during development. Quality-control metadata included low lead standard deviation, extreme amplitude, excessive range, sustained flatness, and limb-lead consistency checks. These flags were recorded for transparency but were not used for automatic exclusion in the frozen primary workflow.

QRS anchoring, median beat, and normal atlas

A QRS-energy anchor rather than a physiological R peak was used. The frozen test detector filtered the geometry signal from 5 to 20 Hz, calculated the mean squared temporal derivative across all 12 leads, smoothed this energy over 80 ms, identified peaks at a height of 10% of the global smoothed-energy maximum with a minimum spacing of 300 ms, and refined each peak to the local energy maximum within ±60 ms. Complete 1.0-second beats from -350 to +650 ms around each anchor were assembled, and the samplewise median across complete beats was used. At least four complete beats were required.

The primary QRS road window was frozen at -120 to +180 ms relative to the QRS-energy anchor. Translation was removed by subtracting the mean 3D road over the first 100 ms of the full aligned beat. The raw orientation-preserving QRS road retained amplitude. A second representation divided the centered QRS road by its own root-mean-square (RMS) radius, the square root of the mean of the squared road norm, to remove global QRS-road scale while preserving orientation.

The normal atlas was derived exclusively from training normals, with one ECG per patient. Of 6,789 selected normal patients, 6,776 contributed successfully aligned QRS roads. At every QRS time point, the atlas stored raw and scale-normalized means and medians, 3×3 covariance matrices with eigenvalue-regularized inverses, and empirical 95th and 99th percentile Euclidean and Mahalanobis envelopes. For covariance regularization, each eigenvalue was floored at the greater of an absolute floor of one trillionth and a relative floor of one millionth of the mean covariance eigenvalue, and the inverse covariance was reconstructed from the regularized eigenspectrum. The same rule was applied to the raw and scale-normalized atlases. No validation or test record contributed to the atlas.

Frozen geometric features

The primary panel contained four prespecified variables: QRS road RMS radius, normalized Euclidean RMS distance from the pointwise normal median road, the fraction of QRS time points whose normalized Mahalanobis distance exceeded the training-normal pointwise 99th-percentile corridor, and mean normalized Mahalanobis distance from the training-normal pointwise mean using the regularized covariance. Raw Euclidean RMS and the normalized Euclidean 99th-percentile corridor fraction were retained as sensitivity variables but were not part of the primary model panel.

Positive magnitude features were log-transformed and standardized using training patient-balanced means and standard deviations. The bounded Mahalanobis 99th-percentile corridor fraction was standardized on its native scale. These transformations were selected a priori according to mathematical scale rather than predictive performance.

Demographic adjustment and model development

Age and sex were included to quantify confounding and incremental value. Exact age was imputed with the training patient-balanced median of 55 years and standardized using the training mean of 53.2749 years and standard deviation (SD) of 17.5309 years. A de-identified age of 90 years or older was retained as an indicator variable. Sex was coded 0 for male and 1 for female. Age- and sex-missing indicators had zero variance in training and were therefore preserved in preprocessing policy but omitted from fitted coefficients.

For each target, three L2-regularized logistic-regression models were fit on the patient-balanced training set: demographic, geometry, and combined. The estimator used C = 1, the lbfgs solver, no class weighting, a maximum of 5,000 iterations, a tolerance of 1e-8, and a fixed random state. Five-fold stratified cross-validation was used only as a development audit; it did not tune hyperparameters or select features. Full-training coefficients were frozen before held-out evaluation. The combined family was prospectively designated primary, with geometry and demographic models retained as comparators.

Held-out validation, software reconstruction, and locked test evaluation

Validation was evaluated without refitting. The combined model showed an area under the receiver operating characteristic curve (AUROC) and average precision (AP) of 0.9997 and 0.9931 for CLBBB, 0.9988 and 0.9585 for CRBBB, and 0.7345 and 0.1014 for IVCD. These results were used only to confirm the pre-test policy: the combined model remained primary for all targets, CLBBB and CRBBB were designated the primary proof-of-concept endpoints, and IVCD was retained as exploratory. There was no recalibration, threshold optimization, feature selection, or model-family switching.

The original source code for one QRS-alignment helper was not persisted. Before any test waveform was read, a deterministic numerical reconstruction was developed using validation-only software regression and then frozen. The selected global rule reproduced 985 of 995 saved validation normalized QRS records exactly under a prespecified numerical tolerance. A small number of validation records remained software-regression exceptions; no record-specific exception rules were introduced. The reconstruction policy was hashed and locked before opening the test fold. This provenance is reported because it is a reproducibility limitation of the present implementation.

After all extractor, atlas, preprocessing, coefficient, and policy hashes passed, fold 10 metadata was opened. All 1,001 test waveforms were then processed using the frozen reconstruction and frozen atlas. Test results were not used to alter any component of the pipeline.

QRS-duration-only sensitivity comparator

To benchmark the geometric models against a duration-only conduction comparator, a separate QRS-energy duration analysis was performed using the same official training, validation, and test folds with deterministic one-ECG-per-patient selection before duration extraction. The comparator used only QRS-energy duration extracted from accepted QRS-energy beats; records with fewer than four usable QRS-energy beats were excluded. Separate one-predictor logistic models were fit for CLBBB, CRBBB, and IVCD against strict normals on the training split and evaluated unchanged on validation and locked test data. This comparator was used as a sensitivity benchmark and did not alter the frozen primary geometric models or endpoint hierarchy.

Morphology-aware conventional ECG comparator

A post hoc morphology-aware conventional 12-lead comparator was additionally evaluated to incorporate lead-specific QRS morphology beyond duration alone. The comparator used the same official training, validation, and test folds and the existing one-ECG-per-patient QRS-duration cohort. Median beats were reconstructed from raw ECG waveforms in millivolts using the frozen QRS-energy anchors. Complete beats from -350 to +650 ms were assembled, with at least four required, and the sample-wise median was calculated. A per-lead local baseline was removed using the first 100 ms of the complete median beat. The morphology QRS window was centered on the QRS-energy anchor and used the previously extracted QRS-energy duration; early and terminal 40-ms windows were defined within this interval.

QRS duration was combined with prespecified morphology descriptors from leads I, V1, V2, V5, and V6. For each lead, features comprised maximum positive QRS amplitude, absolute maximum negative QRS amplitude, R/S amplitude ratio, signed QRS area, absolute QRS area, net-to-absolute QRS area ratio, terminal 40-ms mean amplitude, terminal 40-ms positive, negative, and net areas, and early 40-ms mean amplitude. Together with QRS duration, this yielded 56 model features. The feature definition and modeling policy were frozen before test performance was evaluated. The primary locked-test results were already known when this post hoc comparator was developed. Its feature set was derived from conventional lead-level QRS morphology rather than from observed geometric-model errors, and the complete comparator specification was fixed before comparator test performance was calculated.

Separate L2-regularized logistic-regression models were fit for CLBBB, CRBBB, and IVCD versus strict normals using the training split only. Missing values, if present, were imputed using training-set medians, and continuous features were standardized using training-set parameters. The estimator used C = 1, the lbfgs solver, no class weighting, a maximum of 5,000 iterations, a tolerance of 1e-8, and a fixed random state of 42. Validation served as an audit only; no feature selection, hyperparameter tuning, test refitting, recalibration, or threshold optimization was performed. This model was intended as a transparent morphology-aware conventional-feature comparator rather than a validated automated implementation of complete clinical bundle-branch-block criteria.

Statistical analysis

At evaluation, one deformation-available ECG per patient was selected using the lowest ECG identifier as a deterministic tie-breaker independent of diagnosis, ECG morphology, model output, or predicted probability. A sensitivity analysis repeated evaluation using the highest ECG identifier per patient. Each BBB target was evaluated against strict normals, excluding the other BBB categories. Metrics were the AUROC, AP, AP-to-prevalence lift, Brier score, log loss, mean predicted probability, and calibration-in-the-large error, operationalized as mean predicted probability minus observed prevalence. No probability threshold was selected, so sensitivity and specificity are not reported as primary endpoints. Additional descriptive calibration analyses estimated logistic calibration intercepts and slopes from the frozen combined-model predictions; no recalibration was performed.

Uncertainty was quantified with 2,000 patient-level stratified bootstrap replicates using a fixed random seed of 42. Positive and normal patients were sampled independently with replacement at their observed counts, preserving both outcome classes in every replicate. For paired model comparisons, the same sampled patients were used for both models. Percentile 95% confidence intervals (CIs) are reported. The recovered bootstrap implementation exactly reproduced the previously stored primary bootstrap replicates and confidence intervals and was also used for the paired shared-ECG morphology comparisons.

The statistical and computational analyses were performed in Python within Google Colab. The environment used Python 3.13.15, with NumPy 2.1.3, pandas 2.2.3, SciPy 1.16.3, scikit-learn 1.6.1, Matplotlib 3.10.0, statsmodels 0.15.0, and PyTorch 2.11.0+cpu. The analysis environment ran on Linux (https://www.linux.org/).

## Results

Cohort characteristics and processing reliability

The patient-exclusive cohort included 339 training CLBBB ECGs from 270 patients, 152 CRBBB ECGs from 139 patients, 163 IVCD ECGs from 163 patients, and 7,254 normal ECGs from 6,789 patients. Validation contained 38 CLBBB ECGs from 33 patients, 25 CRBBB ECGs from 23 patients, 20 IVCD ECGs from 20 patients, and 912 normal ECGs from 865 patients. The test fold contained 42 CLBBB ECGs from 33 patients, 22 CRBBB ECGs from 21 patients, 26 IVCD ECGs from 26 patients, and 911 normal ECGs from 834 patients (Tables 1, 2).

**Table 1:** Patient-exclusive cohort flow Counts precede the one-ECG-per-patient model evaluation step. Training uses PTB-XL folds 1-8, validation fold 9, and test fold 10. ECG: electrocardiogram; CLBBB: complete left bundle branch block; CRBBB: complete right bundle branch block; IVCD: intraventricular conduction delay; PTB-XL: Physikalisch-Technische Bundesanstalt - Extra Large version 1.0.3 (National Metrology Institute of Germany, Braunschweig, Germany)

Split	Group	ECGs	Unique patients
Training	CLBBB	339	270
Training	CRBBB	152	139
Training	IVCD	163	163
Training	Normal	7,254	6,789
Validation	CLBBB	38	33
Validation	CRBBB	25	23
Validation	IVCD	20	20
Validation	Normal	912	865
Test	CLBBB	42	33
Test	CRBBB	22	21
Test	IVCD	26	26
Test	Normal	911	834

**Table 2:** Patient-level test demographics Exact ages above 89 years were deidentified in PTB-XL; those records are represented by a separate indicator in modeling. IQR: interquartile range; CLBBB: complete left bundle branch block; CRBBB: complete right bundle branch block; IVCD: intraventricular conduction delay; PTB-XL: Physikalisch-Technische Bundesanstalt - Extra Large version 1.0.3 (National Metrology Institute of Germany, Braunschweig, Germany)

Group	Patients	Age (years), median (IQR)	Age ≥ 90 deidentified, n	Female, n (%)
CLBBB	33	71.0 (67.0-80.0)	4	22 (66.7)
CRBBB	21	72.0 (65.0-77.0)	0	7 (33.3)
IVCD	26	59.5 (50.5-67.5)	0	13 (50.0)
Normal	834	53.0 (40.0-64.0)	0	397 (47.6)

All 1,001 locked test ECGs were processed successfully, achieved alignment, and yielded the frozen deformation features. Median QRS-template correlation was 0.9979 in CLBBB, 0.9946 in CRBBB, 0.9957 in IVCD, and 0.9961 in normals. The median number of complete beats was 12, 10, 9.5, and 10, respectively (Table 3, Figure 1).

**Table 3:** Locked test alignment quality control bpm: beats per minute; CLBBB: complete left bundle branch block; CRBBB: complete right bundle branch block; IVCD: intraventricular conduction delay.

Group	N	Alignment success	Complete beats, median	Heart rate, median (bpm)	RR coefficient of variation, median	Template r, median
CLBBB	42	1.000	12.0	80.3	0.023	0.9979
CRBBB	22	1.000	10.0	69.5	0.027	0.9946
IVCD	26	1.000	9.5	64.2	0.020	0.9957
Normal	911	1.000	10.0	68.3	0.025	0.9961

**Figure 1:**
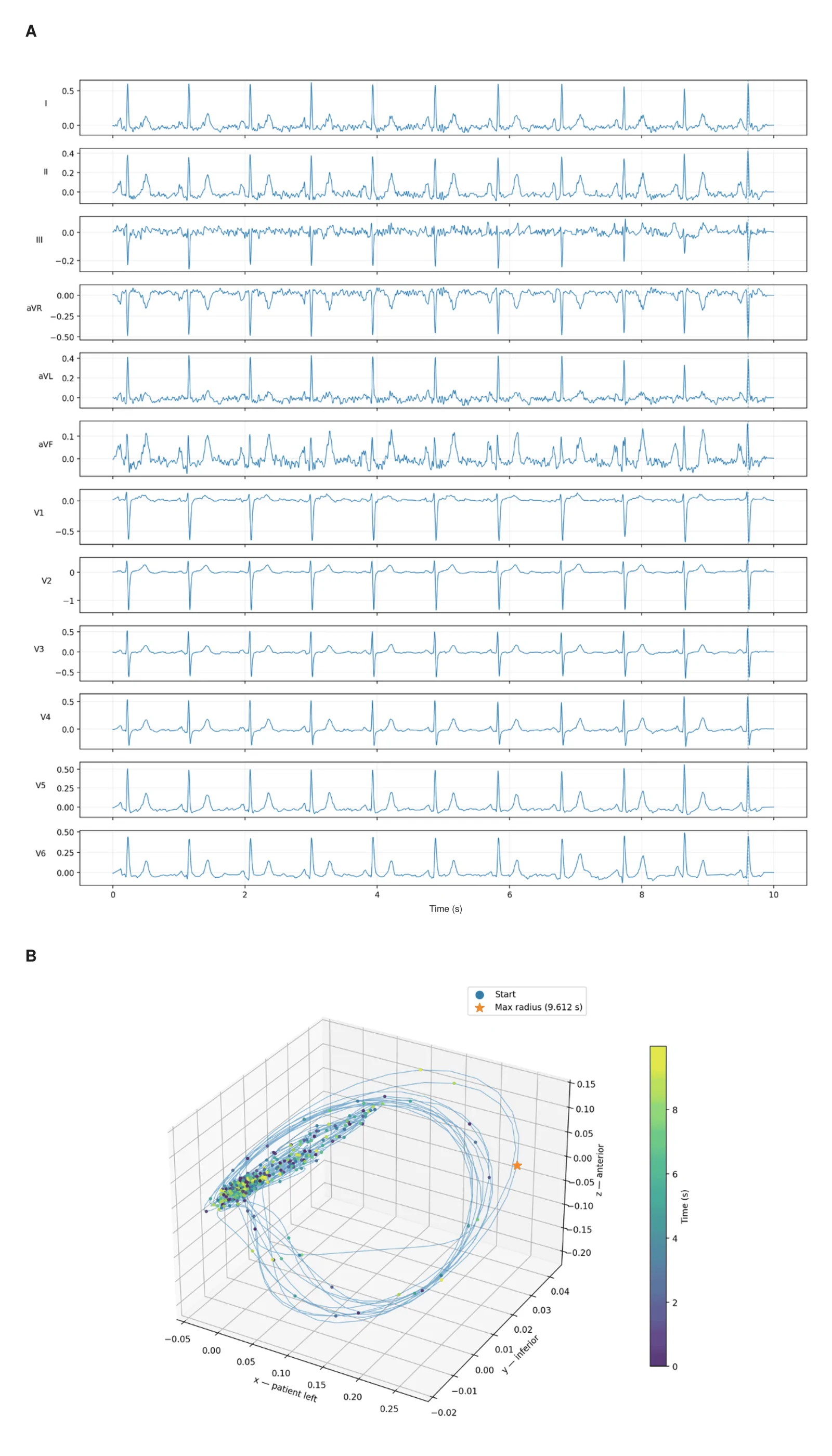
Illustrative construction of the ECG road (A) Geometry-preprocessed 12-lead electrocardiogram filtered at 0.5-40 Hz. The vertical dashed line marks the time of maximum three-dimensional ECG-road radius at 9.612 seconds. (B) Raw-orientation three-dimensional centroid road generated by mapping each lead amplitude to the frozen spatial embedding and averaging the 12 lead-specific spatial points over time. The axes are patient left (x), inferior (y), and anterior (z). The precordial embedding is a research coordinate system, not a validated Frank lead-field reconstruction. ECG: electrocardiogram

Held-out validation

On patient-balanced held-out validation, the combined CLBBB model evaluated 33 cases against 865 normals and achieved an AUROC of 0.9997 (95% CI 0.9991-1.0000) and AP of 0.9931 (0.9796-1.0000). For CRBBB (23 cases, 865 normals), the AUROC was 0.9988 (0.9970-0.9999), and AP was 0.9585 (0.9003-0.9964). IVCD (20 cases, 865 normals) was substantially less separable, with an AUROC of 0.7345 (0.6286-0.8284) and AP of 0.1014 (0.0378-0.2340) (Table 4). Excluding the two patient-balanced CLBBB records carrying a QRS-boundary support flag produced essentially unchanged geometric and combined performance; no CRBBB or IVCD patient-balanced record carried that flag.

**Table 4:** Held-out validation combined-model performance AUROC: area under the receiver operating characteristic curve; AP: average precision; CI: confidence interval; CLBBB: complete left bundle branch block; CRBBB: complete right bundle branch block; IVCD: intraventricular conduction delay

Target	N (positive)	AUROC (95% CI)	AP (95% CI)	Brier	Log loss
CLBBB	898 (33)	0.9997 (0.9991-1.0000)	0.9931 (0.9796-1.0000)	0.0034	0.0119
CRBBB	888 (23)	0.9988 (0.9970-0.9999)	0.9585 (0.9003-0.9964)	0.0056	0.0176
IVCD	885 (20)	0.7345 (0.6286-0.8284)	0.1014 (0.0378-0.2340)	0.0214	0.0996

Locked test discrimination

The deterministic patient-balanced test set contained 914 unique patients. Binary evaluation cohorts contained a common set of 834 normals plus 33 CLBBB cases (n = 867), 21 CRBBB cases (n = 855), or 26 IVCD cases (n = 860). The primary combined model achieved near-perfect discrimination for both canonical bundle branch blocks (Table 5). For CLBBB, the AUROC was 0.9998 (95% CI 0.9992-1.0000) and AP was 0.9951 (0.9833-1.0000), with an AP-to-prevalence lift of 26.14. For CRBBB, the AUROC was 0.9995 (0.9984-1.0000), AP was 0.9858 (0.9546-1.0000), and AP lift was 40.14. 

**Table 5:** Locked test performance by model family The combined model was prospectively designated primary. Other bundle branch block categories were excluded from each target-versus-normal binary evaluation. CIs are from 2,000 patient-level stratified bootstrap replicates. AUROC: area under the receiver operating characteristic curve; AP: average precision; CI: confidence interval; CLBBB: complete left bundle branch block; CRBBB: complete right bundle branch block; IVCD: intraventricular conduction delay

Target	Model	N (positive)	AUROC (95% CI)	AP (95% CI)	Brier	Log loss
CLBBB	Demographic	867 (33)	0.8383 (0.7661-0.8974)	0.2626 (0.1498-0.4182)	0.0333	0.1316
CLBBB	Geometry	867 (33)	0.9996 (0.9985-1.0000)	0.9919 (0.9718-1.0000)	0.0029	0.0117
CLBBB	Combined	867 (33)	0.9998 (0.9992-1.0000)	0.9951 (0.9833-1.0000)	0.0029	0.0107
CRBBB	Demographic	855 (21)	0.8490 (0.7626-0.9214)	0.1548 (0.0883-0.3229)	0.0225	0.0947
CRBBB	Geometry	855 (21)	0.9989 (0.9967-1.0000)	0.9704 (0.9195-1.0000)	0.0048	0.0171
CRBBB	Combined	855 (21)	0.9995 (0.9984-1.0000)	0.9858 (0.9546-1.0000)	0.0040	0.0138
IVCD	Demographic	860 (26)	0.5773 (0.4722-0.6832)	0.0411 (0.0301-0.0820)	0.0294	0.1363
IVCD	Geometry	860 (26)	0.5428 (0.4356-0.6542)	0.0360 (0.0277-0.0622)	0.0296	0.1404
IVCD	Combined	860 (26)	0.5857 (0.4725-0.6982)	0.0443 (0.0311-0.0852)	0.0295	0.1393

IVCD did not reproduce the validation discrimination. The test AUROC was 0.5857 (0.4725-0.6982), and AP was 0.0443 (0.0311-0.0852), only 1.46-fold above the 3.02% prevalence. This negative result was retained without model switching, consistent with the pre-test policy (Figure 2).

**Figure 2:**
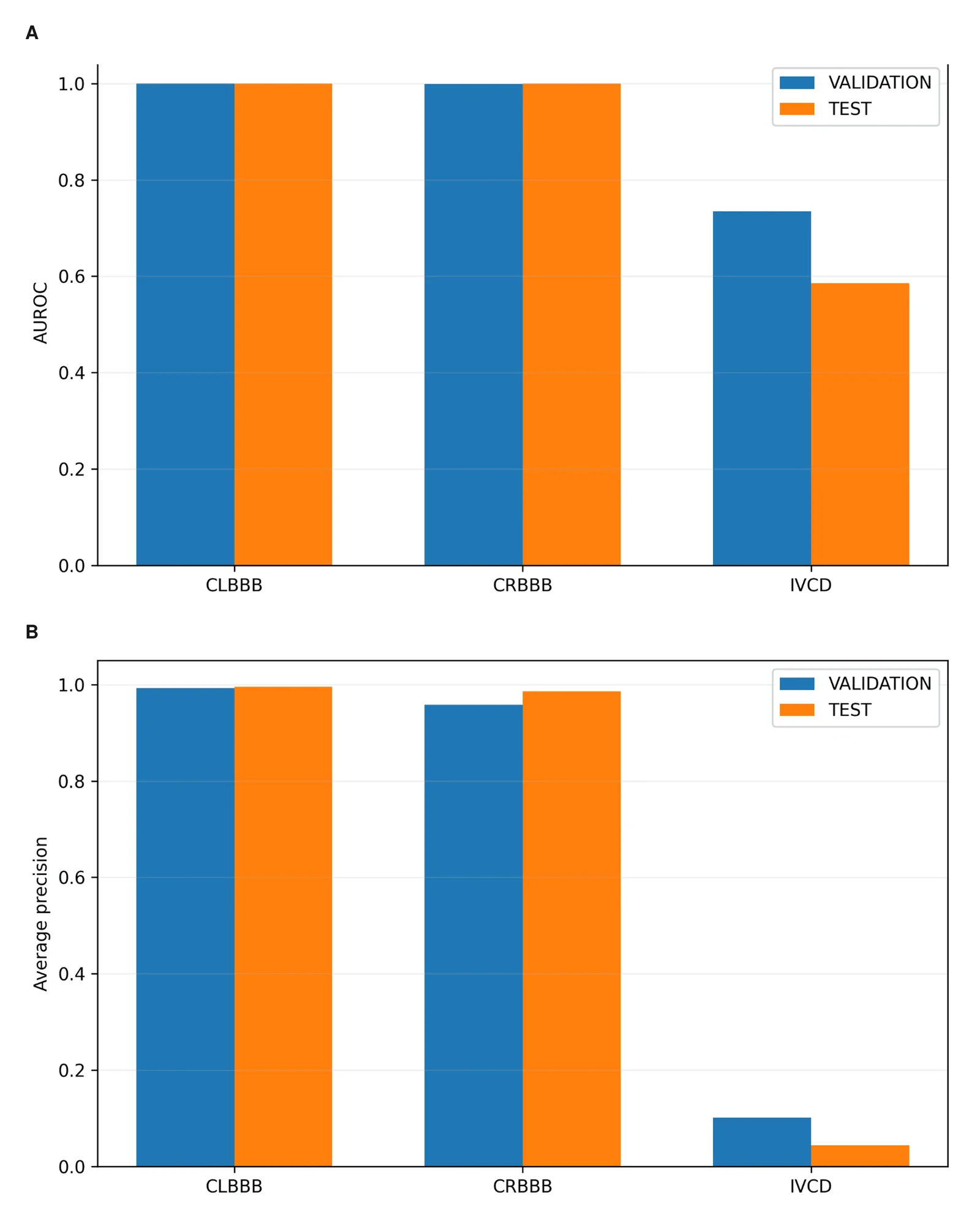
Held-out validation and locked test performance of the combined model (A) Area under the receiver operating characteristic curve (AUROC) on the held-out validation and locked test folds; (B) Average precision on the held-out validation and locked test folds. CLBBB: complete left bundle branch block; CRBBB: complete right bundle branch block; IVCD: intraventricular conduction delay

Geometric phenotype on the locked test fold

The geometric feature distributions explain the contrasting target performance (Table 6). Compared with normals, CLBBB showed both larger road scale and marked normalized deformation: median QRS road RMS radius 0.286 versus 0.138, normalized Euclidean RMS 1.059 versus 0.424, Mahalanobis 99th-percentile corridor fraction 0.298 versus 0, and mean normalized Mahalanobis distance 4.266 versus 1.296. CRBBB showed less change in road scale but strong shape deformation. In contrast, IVCD substantially overlapped normals.

**Table 6:** Locked test geometric phenotype, median (IQR) Patient-balanced locked test data: CLBBB n = 33, CRBBB n = 21, IVCD n = 26, normal n = 834. IQR: interquartile range; QRS: ventricular depolarization complex; RMS: root mean square; CLBBB: complete left bundle branch block; CRBBB: complete right bundle branch block; IVCD: intraventricular conduction delay

Feature	CLBBB	CRBBB	IVCD	Normal
QRS road RMS radius	0.286 (0.260-0.417)	0.151 (0.131-0.176)	0.144 (0.109-0.171)	0.138 (0.119-0.162)
Normalized Euclidean RMS	1.059 (0.954-1.121)	0.959 (0.790-1.141)	0.420 (0.354-0.509)	0.424 (0.346-0.542)
Mahalanobis fraction above the 99th percentile	0.298 (0.252-0.523)	0.238 (0.205-0.517)	0.000 (0.000-0.000)	0.000 (0.000-0.000)
Normalized Mahalanobis mean	4.266 (3.570-5.786)	5.547 (4.394-7.035)	1.319 (1.161-1.697)	1.296 (1.090-1.582)

These patterns suggest that the normal-atlas approach distinguishes canonical bundle-branch activation patterns primarily through trajectory shape rather than through amplitude alone. CLBBB additionally showed a prominent scale shift, while CRBBB was characterized by strong normalized deformation despite a near-normal median road radius.

Incremental value of geometry

For CLBBB, the geometry-only model achieved a test AUROC of 0.9996 and AP of 0.9919, compared with 0.8383 and 0.2626 for the demographic model. For CRBBB, the geometry model achieved an AUROC of 0.9989 and AP of 0.9704 versus 0.8490 and 0.1548 for the demographic model. For IVCD, no paired comparison demonstrated a clear improvement of the combined model over either comparator (Table 7). Together, these prespecified comparisons indicate that the strong CLBBB and CRBBB discrimination was not explained by age and sex; they do not establish incremental diagnostic value over conventional ECG morphology.

**Table 7:** Paired locked test differences for the combined model AUROC: area under the receiver operating characteristic curve; CI: confidence interval; CLBBB: complete left bundle branch block; CRBBB: complete right bundle branch block; IVCD: intraventricular conduction delay.

Target	Comparison	Metric	Difference	95% CI
CLBBB	vs demographic	AUROC	+0.1615	+0.1025 to +0.2335
CLBBB	vs demographic	Average precision	+0.7325	+0.5769 to +0.8422
CLBBB	vs geometry	AUROC	+0.0002	-0.0001 to +0.0010
CLBBB	vs geometry	Average precision	+0.0032	-0.0035 to +0.0152
CRBBB	vs demographic	AUROC	+0.1505	+0.0784 to +0.2374
CRBBB	vs demographic	Average precision	+0.8310	+0.6682 to +0.9010
CRBBB	vs geometry	AUROC	+0.0006	+0.0000 to +0.0019
CRBBB	vs geometry	Average precision	+0.0155	0.0000 to +0.0435
IVCD	vs demographic	AUROC	+0.0084	-0.0547 to +0.0738
IVCD	vs demographic	Average precision	+0.0032	-0.0231 to +0.0296
IVCD	vs geometry	AUROC	+0.0429	-0.0232 to +0.1014
IVCD	vs geometry	Average precision	+0.0083	-0.0021 to +0.0297

QRS-duration-only sensitivity comparator

The QRS-duration-only comparator successfully extracted duration features from 9,056 of 9,081 one-record-per-patient ECGs (99.7%); the 25 failures were all due to fewer than four usable QRS-energy beats. Because the duration-only analysis reconstructed its one-ECG-per-patient cohort independently from PTB-XL metadata, target records were required to contain exactly one positive conduction-diagnostic code (CLBBB, CRBBB, or IVCD), with myocardial infarction, ST/T-change, and hypertrophy classes excluded before selection of the lowest eligible ECG identifier per patient. After duration extraction, the test cohort contained 29 CLBBB, 11 CRBBB, 27 IVCD, and 836 normal patients, compared with 33, 21, 26, and 834, respectively, in the primary geometry analysis. In the usable locked test set, duration alone performed strongly for CLBBB, moderately for CRBBB, and weakly for IVCD (Table 8). QRS duration alone therefore provided a useful secondary sensitivity benchmark but did not constitute a complete conventional ECG comparator because clinical bundle-branch-block interpretation also incorporates lead-specific morphology. 

**Table 8:** QRS-duration-only secondary sensitivity comparator on the locked test set This sensitivity model used QRS-energy duration as its only predictor after deterministic one-ECG-per-patient selection. QRS-duration extraction succeeded in 9,056 of 9,081 records; 25 failed because fewer than four usable QRS-energy beats were available. AP: average precision; AUROC: area under the receiver operating characteristic curve; CI: confidence interval; CLBBB: complete left bundle branch block; CRBBB: complete right bundle branch block; IVCD: intraventricular conduction delay; QRS: ventricular depolarization complex

Target	Usable test cohort	Median QRS duration, target vs normal (ms)	AUROC (95% CI)	AP (95% CI)	Brier	Log loss
CLBBB	29 vs 836 (n = 865)	196 vs 138	0.9942 (0.9815-1.0000)	0.9713 (0.9134-1.0000)	0.0030	0.0135
CRBBB	11 vs 836 (n = 847)	150 vs 138	0.8498 (0.6650-0.9940)	0.5420 (0.2819-0.8241)	0.0075	0.0418
IVCD	27 vs 836 (n = 863)	142 vs 138	0.6329 (0.5319-0.7261)	0.0509 (0.0358-0.0849)	0.0302	0.1384

Morphology-aware conventional ECG comparator

The morphology-aware conventional comparator showed near-ceiling locked-test discrimination for canonical bundle branch block. For CLBBB (29 cases and 836 normals), AUROC was 1.0000 (95% bootstrap CI 1.0000-1.0000) and AP was 1.0000 (1.0000-1.0000). For CRBBB (11 cases and 836 normals), AUROC was 0.9998 (0.9990-1.0000), and AP was 0.9860 (0.9416-1.0000). IVCD remained substantially less separable (27 cases and 836 normals), with AUROC 0.6614 (0.5508-0.7611) and AP 0.0935 (0.0469-0.2163).

Because the primary combined-model and morphology-aware analyses had slightly different evaluable cohorts, a sensitivity analysis restricted both models to identical ECGs. The shared cohorts contained 29 CLBBB cases and 834 normals (n = 863), 11 CRBBB cases and 834 normals (n = 845), and 25 IVCD cases and 834 normals (n = 859). For CLBBB, combined-model versus morphology-aware AUROC was 0.9998 versus 1.0000, and AP was 0.9937 versus 1.0000. For CRBBB, AUROC was 0.9991 versus 0.9998, and AP was 0.9564 versus 0.9860. For IVCD, AUROC was 0.5931 versus 0.6523, and AP was 0.0442 versus 0.0934.

Paired morphology-minus-combined-model AUROC differences were +0.0002 (95% CI 0.0000 to 0.0009) for CLBBB, +0.0007 (-0.0009 to 0.0028) for CRBBB, and +0.0592 (-0.0773 to 0.1925) for IVCD. Corresponding AP differences were +0.0063 (0.0000 to 0.0213), +0.0296 (-0.0445 to 0.1266), and +0.0493 (-0.0090 to 0.1814), respectively. All paired 95% bootstrap intervals included or touched zero (Table 9). For CLBBB, the zero lower bound reflects the ceiling performance of the morphology comparator, which precluded negative morphology-minus-combined-model differences in many bootstrap samples. The CRBBB shared-ECG comparison contained only 11 positive cases and should therefore be interpreted cautiously.

**Table 9:** Paired shared-ECG comparison of the primary combined model and morphology-aware conventional comparator Differences are defined as morphology-aware comparator minus primary combined model. Cls are percentile 95% intervals from 2,000 patient-level stratified paired bootstrap replicates using the same sampled patients for both models. No model refitting, tuning, recalibration, or threshold optimization was performed. AUROC: area under the receiver operating characteristic curve; AP: average precision; CI: confidence interval; CLBBB: complete left bundle branch block; CRBBB: complete right bundle branch block; IVCD: intraventricular conduction delay

Target and shared cohort	Combined AUROC	Morphology AUROC	Δ AUROC, morphology − combined (95% CI)	Combined AP	Morphology AP	Δ AP, morphology − combined (95% CI)
CLBBB, n=863 (29 positive, 834 normal)	0.9998	1.0000	+0.0002 (0.0000 to 0.0009)	0.9937	1.0000	+0.0063 (0.0000 to 0.0213)
CRBBB, n=845 (11 positive, 834 normal)	0.9991	0.9998	+0.0007 (-0.0009 to 0.0028)	0.9564	0.9860	+0.0296 (-0.0445 to 0.1266)
IVCD, n=859 (25 positive, 834 normal)	0.5931	0.6523	+0.0592 (-0.0773 to 0.1925)	0.0442	0.0934	+0.0493 (-0.0090 to 0.1814)

Patient-selection sensitivity and calibration

Selection of the highest rather than lowest ECG identifier changed the selected record for 71 of 914 patients but produced essentially unchanged combined-model discrimination. For CLBBB, AUROC/AP changed from 0.9998/0.9951 to 0.9998/0.9960; for CRBBB, from 0.9995/0.9858 to 0.9996/0.9872; and for IVCD, from 0.5857/0.0443 to 0.5838/0.0445.

For the original combined models, mean predicted probability minus observed prevalence was -0.0041 for CLBBB, -0.0031 for CRBBB, and -0.0072 for IVCD. Descriptive logistic calibration intercepts/slopes were 2.436/1.469 for CLBBB, 2.581/2.701 for CRBBB, and -2.045/0.359 for IVCD. These jointly estimated calibration intercepts are distinct from calibration-in-the-large, which was defined as mean predicted probability minus observed prevalence. The near-ceiling discrimination for CLBBB and CRBBB limits the stability and interpretability of the fitted calibration slopes, whereas the IVCD slope below 1 was consistent with predictions that were too extreme relative to the observed outcomes. These calibration analyses were descriptive; no recalibration was applied.

## Discussion

This patient-exclusive, prospectively locked study provides a focused proof of concept for representing the 12-lead ECG as a time-resolved 3D trajectory and quantifying its deformation relative to a training-normal atlas. Four findings are central. First, the frozen geometric panel generalized with near-perfect held-out test discrimination for clean CLBBB and CRBBB. Second, geometry-only models performed almost identically to the prespecified combined models and far better than demographic models, indicating that the signal was carried by ECG-derived geometric features rather than by age or sex imbalance. Third, a post hoc morphology-aware conventional 12-lead comparator also showed near-ceiling discrimination for CLBBB and CRBBB, and paired analyses restricted to identical ECGs did not demonstrate a clear performance advantage for either representation. Fourth, IVCD performance was weak on the locked test fold despite moderate validation performance, emphasizing that the method did not simply produce uniformly high scores for every conduction label.

The feature-level patterns are consistent with the study hypothesis. CLBBB produced both increased trajectory scale and large normalized deformation, whereas CRBBB produced strong normalized deformation with much less scale change. These findings are consistent with prior work showing that CLBBB has distinct ECG criteria and altered left ventricular endocardial activation [14,15]. That distinction is appealing because it separates amplitude and scale from shape, a central motivation for the dual raw and scale-normalized atlas representations. IVCD, by contrast, overlapped the normal atlas on the same four features. This may reflect the clinical heterogeneity of nonspecific intraventricular delay, which can arise from diverse activation patterns and may not share one dominant trajectory deformation.

The work is related to, but deliberately not equivalent to, vectorcardiography. Vectorcardiography represents cardiac electrical forces in orthogonal spatial leads and has a long history in conduction analysis [2,6,7]. Contemporary work has shown that Frank-like vectorcardiograms can be reconstructed from standard ECGs with regression or inverse transformations and can retain clinically useful spatial information [3-5]. Our framework instead begins with a fixed lead-vector research embedding and studies the geometry of all 12 embedded lead points and their centroid over time. This creates a convenient mathematical object for local differential geometry, covariance, constellation shape, and normal-atlas residuals. The distinction matters: the present precordial vectors are not validated physical lead fields, and the strong classification results should not be interpreted as validation of the embedding’s anatomical fidelity.

The study also differs from high-capacity deep neural ECG classifiers. PTB-XL has enabled powerful end-to-end models, and recent explainability work has shown that left BBB predictions often depend on clinically expected QRS regions and durations [11,12]. Our aim was not to maximize classification accuracy against those systems, but to test whether a compact, inspectable set of geometric variables could express a disease-related deformation field. The near-equivalence of four geometry variables to the combined model for CLBBB and CRBBB supports that representation-level objective. The duration-only sensitivity analysis showed that CLBBB was almost fully captured by QRS duration, whereas CRBBB showed substantially lower duration-only performance than geometric performance. However, the morphology-aware conventional comparator also achieved near-ceiling discrimination for both canonical BBBs, indicating that lead-specific conventional morphology contains much of the same label-relevant information.

The magnitude of the CLBBB and CRBBB performance should therefore be interpreted cautiously. These are canonical ECG diagnoses whose labels are assigned from the ECG itself, and the clean proof-of-concept cohort deliberately excluded major competing diagnostic superclasses. High discrimination consequently demonstrates that the experimental geometric transformation preserves ECG information relevant to these labels; it does not establish clinical superiority over conventional 12-lead morphology. In the shared-ECG comparison, the morphology-aware conventional and primary combined models produced very similar discrimination, and all paired 95% bootstrap CIs for AUROC and average-precision differences included or touched zero. The present data therefore support representational validity within PTB-XL rather than added diagnostic necessity or clinical superiority. A stronger next study should ask whether geometric deformation adds value in harder tasks, including atypical or incomplete blocks, mixed pathology, myocardial infarction in the presence of BBB, cardiac resynchronization therapy response phenotyping, myocardial scar characterization, or ventricular arrhythmic risk.

The locked workflow is an important strength. The normal atlas, transformations, logistic coefficients, primary model family, and endpoint hierarchy were fixed before the test fold was opened; all 1,001 test waveforms were processed successfully; and no post-test refitting, recalibration, threshold optimization, feature selection, or model switching occurred in the primary analysis. The preservation of the negative IVCD result is particularly important because it demonstrates that the analysis plan was not altered to rescue a weaker endpoint. The subsequent morphology-aware comparator, alternative ECG-selection sensitivity analysis, bootstrap reproducibility audit, and calibration assessment did not modify the original frozen models or primary locked-test results.

Limitations

Several limitations require emphasis. First, the study uses a single public dataset and therefore provides internal held-out rather than external validation; dataset-specific acquisition, labeling, and population characteristics may contribute to the results. Second, the experimental precordial lead-vector embedding has not been validated against measured Frank vectorcardiography or body-surface lead fields, and sensitivity analyses using established Kors or inverse Dower transforms are needed [3,4]. Third, the original source of one QRS-alignment helper was not persisted; a deterministic reconstruction was numerically validated on held-out validation and frozen before the test fold, but it did not exactly reproduce 10 of 995 saved validation roads, a gap that should be eliminated in a prospective reimplementation. Fourth, positive test counts were modest, particularly for CRBBB, resulting in near-ceiling but sample-limited performance estimates. Fifth, the clean-versus-normal design does not represent the full clinical differential diagnosis. Sixth, age and sex differed across groups; although prespecified demographic comparators and combined adjustment were used, no matching or causal adjustment was performed.

Seventh, the diagnostic labels are themselves derived from ECG findings, creating dependence between the information available to the models and the reference labels. Accordingly, the high CLBBB and CRBBB discrimination should be interpreted as evidence that the geometric representation retains label-relevant ECG morphology rather than as independent validation of diagnostic superiority or clinical utility. Eighth, although the morphology-aware comparator incorporates QRS duration and prespecified lead-specific amplitude, polarity, and area features, it is a transparent conventional-feature benchmark rather than a validated automated implementation of complete clinical BBB criteria or commercial ECG interpretation. Finally, PTB-XL does not provide the independent imaging, electrophysiologic, or outcome reference standards needed to determine whether the geometric representation adds clinically useful information for myocardial scar, cardiac resynchronization therapy response, ventricular arrhythmia risk, or related endpoints.

These limitations define a clear development path. Future validation should use a prospectively preserved implementation, derive established XYZ or Frank representations in parallel, and evaluate the same normal-atlas deformation metrics in external ECG cohorts. A clinically meaningful extension would test whether geometric residual fields add information for ischemic injury, ventricular substrate, cardiac resynchronization therapy response, or arrhythmic outcomes when conventional scalar ECG interpretation is limited. The broader 12-point constellation and differential-geometric components of the framework may be most useful in those more complex settings, where they can quantify spatial coherence and local departures from a normal activation manifold rather than merely recapitulate an established QRS pattern.

## Conclusions

A compact, orientation-preserving 3D ECG road and training-normal QRS deformation atlas showed strong held-out discrimination of clean CLBBB and CRBBB in PTB-XL, while showing limited discrimination of heterogeneous IVCD on the locked test fold. A morphology-aware conventional 12-lead comparator showed similarly near-ceiling discrimination for CLBBB and CRBBB, and paired analyses on identical ECGs did not demonstrate a clear performance advantage for either representation.

The findings support the ECG-road concept as an interpretable mathematical representation of canonical conduction-pattern information already present in the 12-lead ECG, not as a clinically validated replacement for standard ECG criteria or evidence of added clinical utility. External validation, established ECG-to-XYZ sensitivity analyses, and comparison with validated conventional conduction criteria and independent clinical or imaging endpoints are necessary next steps.
